# Safety and efficacy of bevacizumab combined with R-CHOP regimen in seven Chinese patients with untreated diffuse large B-cell lymphoma

**DOI:** 10.1186/1475-2867-14-5

**Published:** 2014-01-18

**Authors:** Zhiying Fu, Jun Zhu, Wen Zheng, Weiping Liu, Zhitao Ying, Yan Xie, Xiaopei Wang, Ningjing Lin, Meifeng Tu, Lingyan Ping, Lijuan Deng, Chen Zhang, Ning Ding, Yuqin Song

**Affiliations:** 1 Key Laboratory of Carcinogenesis and Translational Research (Ministry of Education), Department of Lymphoma, Peking University Cancer Hospital & Institute, Beijing 100142, China

**Keywords:** Bevacizumab, DLBCL, Safety, Efficacy

## Abstract

**Background:**

Rituximab plus CHOP (R-CHOP) significantly improved the outcome of diffuse large B cell lymphoma (DLBCL), a common sub-type of non-Hodgkin lymphoma. But 40% – 50% of DLBCL patients cannot be cured by this regimen. Some clinical trials showed that bevacizumab might be useful in the treatment of DLBCL. This study evaluated the safety and efficacy of bevacizumab combined with the R-CHOP (A-R-CHOP) regimen in Chinese patients with previously untreated DLBCL.

**Methods:**

Patients with previously untreated DLBCL received A-R-CHOP regimen therapy. All patients with complete response (CR)/ unconfirmed complete response(CRu) after 8 cycles of A-R-CHOP received the bevacizumab maintenance therapy once every 3 weeks. The remained bulky disease was treated with radiotherapy.

**Results:**

Seven Chinese patients were treated. All of them had bulky diseases. One patient had progressive disease after 4 cycles of A-R-CHOP therapy. The rest six patients completed 8 cycles of A-R-CHOP treatment. All of these six patients reached CR/CRu (5 CR, 1 CRu). Bevacizumab maintenance therapy was given to 4 CR patients. All 7 patients experienced Grade 3/4 hematologic adverse events; additionally, one had Grade 3 gastrointestinal toxicity and one had Grade 1 epistaxis. During bevacizumab maintenance therapy, one patient had Grade 1 gingival bleeding, another experienced Grade 1 proteinuria and then Grade 3 congestive heart failure 4 months after completion of maintenance therapy. At the end of July 2013, the patient who had progressive disease after 4 cycles of A-R-CHOP died of progressive disease, the other six remained CR response.

**Conclusions:**

The A-R-CHOP regimen is effective for untreated DLBCL, but may cause bevacizumab-specific toxicities, which should be monitored.

## Introduction

According to statistics data in 2008, non-Hodgkin’s lymphoma (NHL) is the 10^th^ most common tumor worldwide, with about 300,000 new cases each year [1]. Diffuse large B cell lymphoma (DLBCL) is one of the most frequently diagnosed sub-types of NHL, which accounts for about 30% – 50% of all NHL [2,3]. Although several global trials (e.g. GELA LNH98.5 [4], RICOVER-60 [5], MInT [6]) have demonstrated the efficacy and safety of rituximab combined with chemotherapy as the standard treatment for DLBCL, 40% – 50% of DLBCL patients cannot be cured by the standard R-CHOP regimen(rituximab, cyclophosphamide, doxorubicin, vincristine and prednisone) [2,3].

The role of the vascular endothelial growth factor (VEGF) in the proliferation of lymphatic malignancies has been tested in several pre-clinical studies [7,8]. VEGF expressed in neoplastic cells of aggressive lymphomas, including DLBCL, mantle cell lymphoma (MCL) and peripheral T-cell lymphoma (PTCL) as well as in indolent lymphomas such as chronic lymphocytic leukemia/small lymphocytic lymphoma [9]. These data suggested that anti-VEGF agents might be useful in the treatment of DLBCL.

Bevacizumab, a 93% humanized IgG1 anti-VEGF monoclonal antibody, has been tested both as a single agent (SWOG 0108 study) [10,11] and in combination with chemotherapy (SWOG0515 study) [12] in DLBCL patients. The preliminary data showed that bevacizumab was generally well tolerated and effective in DLBCL. But they were just single-arm phase II trials. Particularly, no phase II studies ever included any Chinese patient.

Based on the preliminary results of the above two phase II studies, a multi-center, randomized, double-blind, placebo-controlled phase III trial (BO20603) was initiated in 2009 to compare the safety and efficacy of bevacizumab plus rituximab and CHOP (A-R-CHOP) versus R-CHOP in previously untreated CD20^+^ DLBCL. Patients were randomized 1:1 to receive A-R-CHOP or R-CHOP treatments. Our cancer center was the top recruiting site in China. In total ten patients were enrolled into this trial at our center—7 in the A-R-CHOP arm and 3 in the R-CHOP arm. This paper reported our own experiences of these 7 Chinese patients who were treated with bevacizumab plus R-CHOP.

## Methods

### Patients

The study was approved by the Ethical Committee of the Peking University Cancer Hospital. The main inclusion criteria were: histologically confirmed DLBCL, left ventricular ejection fraction (LVEF) ≥ 50%, absolute neutrophils count **(**ANC) ≥ 1,500/μL, platelet count ≥ 100,000/μL, adequate hepatic function (total bilirubin < 1.5 × upper limit of normal [ULN], both AST and ALT < 2.5 × ULN), adequate renal function (serum creatinine < 2 mg/dL or 177 μmol/L), and urine dipstick test for proteinuria < 2+. The main exclusion criteria were: CNS involvement, major blood vessel invasion per CT-scan, and clinical, radiological, or endoscopic evidence of lymphoma infiltration of the gastrointestinal tract.

### Pathological diagnosis

The DLBCL were classified according to the 4^th^ Edition of World Health Organization (WHO) Classification of Tumours of Haematopoietic and Lymphoid Tissues [13]. The diagnoses were confirmed with both morphology and immunohistochemistry (IHC).The IHC tests included those for leucocyte antigen, CD45RO (UCHL1), CD3, CD5, CD7, CD20 (L26), CD10, BCL6, MUM1, CD79, CD19, CD30, CD15, CD68 and EMA.

### Laboratory evaluation and tumor staging

The DLBCL was staged according to the Ann Arbor staging system. The workup included hematology, blood sedimentation, lactate dehydrogenase, beta 2-micro globulin, C-reaction protein, routine urine, CT scans of neck, chest cavity, abdominal cavity and pelvic cavity, ultrasound cardiogram (UCG) and bone marrow smear.

### Treatments

Patients received the A-R-CHOP regimen as an induction therapy. The first tumor response assessment was taken after 4 cycles of treatment. Patients who reached CR/CRu or PR at this time continued to receive another 4 cycles of the A-R-CHOP regimen.

The second response assessment was performed after 8 cycles of A-R-CHOP therapy. Patients who achieved CR/CRu received bevacizumab monotherapy (15 mg/kg, every 3 weeks) as a maintenance therapy. The interval between the last dose of bevacizumab in A-R-CHOP induction therapy and the first dose of the maintenance therapy could not exceed 8 weeks. The details of the A-R-CHOP regimen were summarized in Table 1.

**Table 1:** The A-R-CHOP regimen*

**Drug**	**Dose**	**Mode**	
Bevacizumab†	15 mg/kg	i.v.	D1
Rituximab‡	375 mg/m^2^	i.v.	D1
Cyclophosphamide	750 mg/m^2^	i.v.	D1
Doxorubicin	50 mg/m^2^	i.v.	D1
Vincristine	1.4 mg/m^2 ^(≤2 mg)	i.v.	D1
Prednisone	100 mg/d	p.o.	D1-5

*1 cycle per 21 days †bevacizumab was given prior to rituximab, the infusion time of bevacizumab is one hour. ‡the infusion time of rituximab is five hours.

After the second tumor assessment, patients who had the bulky disease (longest diameter ≥ 7.5 cm by CT scan, as defined by protocol) could receive the concomitant radiation therapy to the bulky lesions during the maintenance treatment phase if they obtain CR/CRu.

No premedication was given before bevacizumab. Dexamethasone (5 mg i.v.) and promethazine (12.5 mg i.m.) were used before rituximab to prevent allergic reactions. Routine prophylactic medications were given before the each cycle of CHOP chemotherapy. Medications included hydration, alkalinization, diuresis, and tropisetron before each cycle. All patients received granulocyte colony stimulating factor as prophylaxis for neutropenia.

### Assessment of response and adverse events

Assessment of response was performed according to NCI-WG 1999 criteria [14].

The safety assessment was performed according to the National Cancer Institute Common Toxicity Criteria for Adverse Events (CTCAE) version 3.0 [15].

### Follow-up

LVEF was measured at the following time points: baseline, after 4 and 8 cycles of therapy, and at 1 year post the first dose of the study treatment. Patients are required to come back to the cancer center every 3 months for follow-up visits. Lab tests and examinations at the follow-up visit include CT scan, hematology, chemistry, and C-reactive protein, and LVEF in patients who experienced cardiac toxicities.

## Results

### Efficacy assessment

From May 2009 to May 2010, seven untreated Chinese DLBCL patients (6 females and 1 male) were enrolled into the A-R-CHOP arm of this trial at our center. Informed consents were obtained from all participants. The median age at initial diagnosis was 34 years (range: 18–50). All patients had bulky disease. Patient characteristics are summarized in Table 2.

**Table 2:** Clinical characteristics of 7 patients with diffuse large B cell lymphoma

**Characteristic**	**n (%)**
Middle Age (range)	34 years old (18-50)
Gender	
Male	1 (14.3)
Female	6 (85.7)
Clinical staging	
II	2 (28.6)
IV	5 (71.4)
Performance status score (Eastern Cooperative Oncology Group)	
0	5 (71.4)
1	1 (14.3)
2	1 (14.3)
International prognostic index	
0-1	1 (14.3)
2	4 (57.1)
3	2 (28.6)
Bulky disease (longest diameter ≥ 7.5 cm by CT scan)	
Mediastinum	5 (71.4)
Abdominal cavity	2 (28.6)
B symptoms	
Weight lose	2 (28.6)
Fever	1 (14.3)
Laboratory abnormalities	
LDH (>240 IU/L)	6 (85.7)
C-reactive protein increased	6 (85.7)

Seven patients received bevacizumab every 21 days (15 mg/kg), with R-CHOP on the first day. After 4 cycles of A-R-CHOP treatment, five patients reached CRu, one PR, and one progressed.

This progressed female patient withdrew from this study and then was treated with DICE regimen but failed again. She died of tumor progression. Her overall survival time was 8 months.

The other six patients completed all 8 cycles of A-R-CHOP therapy. The second assessment showed five CR and one CRu. Among these six patients, two female patients did not receive the bevacizumab maintenance treatment. One patient with CR did not receive maintenance treatment because the planned maintenance therapy was delayed by radiotherapy and not started within 8 weeks after the last dose of bevacizumab in A-R-CHOP. The other one was stopped by the sponsor of the trial due to the risk-benefit assessment. The median number of total bevacizumab treatments was 12 times. The median number of maintenance treatments was 4 times. All six patients were alive and remained in CR when the follow-up ended on 30 July 2013 (Table 3).

**Table 3:** Treatment outcome of all seven patients

**Number**	**1**^**st **^**assessment after cycles 1-4**	**2**^**nd **^**assessment after cycles 5-8**	**Bevacizumab maintaining times**	**Radiotherapy (Gy)**	**3**^**rd **^**assessment**
1	CRu	CR	0	40.6	CR
2	CRu	CR	7	40	CR
3	CRu	CR	7	36	CR
4	PD	NA^a^	0	No	NA
5	CRu	CR	6	42	CR
6	CRu	CR	4	42	CR
7	PR	CRu	0	50	CR

CR: complete remission, CRu: complete remission unconfirmed, PR: partial remission, PD: progressive disease. NA, not assessed. ^a^Patient No. 4 withdrew after the first assessment.

### Safety

All seven patients experienced Grade 3/4 hematologic toxicities. One had Grade 3GI toxicity and Grade 2 thrombocytopenia. Another patient suffered from the Grade 1 epistaxis. In the maintenance treatment phase, one patient had transient gum bleeding, another one experienced Grade 1 proteinuria, and later developed Grade 3 congestive heart failure (CHF) after having completed the bevacizumab maintenance therapy.

## Discussion

Bevacizumab was the first anti-angiogenic drug that was approved by the U.S. Food and Drug Administration in 2004 and by the European Medicines Agency in 2005. Several randomized trials have demonstrated that adding bevacizumab into standard chemotherapies could prolong the overall survival in colorectal cancer and progression-free survival in non-small cell lung cancer, breast cancer and renal cell carcinoma. However, the safety concerns of the administration of bevacizumab have existed for a long time. E.g. a meta-analysis suggested that the bevacizumab was associated with a higher risk for all grade 3/4 AE [16].

For the treatment of lymphoma, bevacizumab had been explored for DLBCL, PTCL, MCL and NK/T cell lymphoma. Study E2404 showed that despite a higher overall response rate, the A-CHOP regimen failed to result in durable remissions and was associated with significant toxicities [17]. For DLBCL, the final result of the phase II study S0515 demonstrated that the A-R-CHOP regimen was not promising in terms of PFS and induced severe toxicities (81% of all 64 patients had Grade 3 or higher toxicities, notably the cardiac toxicities and gastrointestinal perforations) [18]. The phase III study (BO20602) was also terminated prematurely due to the risk-benefit assessment that was performed by the independent Data and Safety Monitoring Board (DSMB) on 30 May 2010. There were several articles discussed the bevacizumab treatment in NHL before, but none of them involved Chinese patients. So the safety and efficacy data of bevacizumab in Chinese lymphoma population are very limited. Although only a very small population was reported in this paper, the information was valuable and could be helpful for lymphoma specialists. In the following part of this section, based on the bevacizumab associated toxicities discovered in this study at our site, we would go to discuss hemorrhage, proteinuria and cardiovascular toxicities.

### Hemorrhage

VEGF is important to maintain the integrity of the vascular endothelial cells. Antagonist of VEGF could reduce the endothelial regeneration and may cause bleeding. A meta-analysis of 12,617 patients who were treated with bevacizumab demonstrated significantly increased risk of bleeding (*RR*: 2.48; 95% CI: 1.93–3.18). The incidence of all-grade hemorrhage was 30.4% (95% CI 21.5-40.9), with 3.5% (95% CI 2.2-5.7%) being high grade (grade 3-5) [19]. To reduce the risk of severe hemorrhage in our study, the protocol excluded patients who have lesions involving large blood vessels on CT scan. Two patients experienced Grade 1 mucosal bleeding: epistaxis (7 cycles of bevacizumab, total dose: 7350 mg), and transient gum bleeding (10 cycles of bevacizumab, total dose: 8850 mg).

### Proteinuria

Proteinuria is associated with bevacizumab treatment [20]. A meta-analysis of 6482 patients treated with bevacizumab showed increased risk of proteinuria (*RR*: 2.79; 95% CI: 1.31–5.95, *P* < 0.001) [21]. Eremina et al. performed renal biopsies on six patients of bevacizumab treatment with proteinuria. The results of these biopsies indicated the glomerular thrombosis microvascular disease in the kidney. The animal experiments showed that bevacizumab could inhibit the protective effect of VEGF on endothelial cell. This inhibition might increase the glomerular filtration permeability and decrease absorption capacity heavily. Eventually the proteinuria appeared [22].

In our study, all seven patients received urine dipstick tests to monitor proteinuria before and during bevacizumab treatment. One patient had asymptomatic proteinuria (urine protein 2+) after 12 bevacizumab treatments (total dose: 10,980 mg). The 24-hour urinary protein quantity in this patient was 248 mg after the proteinuria was 3+ in the dipstick test. There was not any significant organic disease found in the kidney ultrasound examination. This patient was under close observation without any treatment. His proteinuria recovered 6 months after its initial diagnosis.

### Cardiovascular toxicities

CHF and myocardial ischemia are most severe cardiovascular toxicities of bevacizumab. One article reported the molecular mechanisms of cardiovascular toxicity of bevacizumab [23]. A number of studies showed that anthracycline-based drug therapy and/or thoracic radiotherapy are risk factors of CHF [24]. Vaklavas et al. reviewed all prospective phase I–III clinical trials published about approved bevacizumab therapies and relevant literature up to December 2008, and found Grade 3–4 left ventricular systolic dysfunction was noted in 0.3% of patients [25].

In our study, a 45-year-old female patient experienced CHF after the following therapies: 8 cycles of A-R-CHOP treatment and 4 cycles of bevacizumab maintenance therapy with concomitant radiotherapy on the bulky abdominal lesion. She did not have any prior history of heart disease. The baseline LVEF was 65%. But it decreased to 35% 4 months after the last dose of bevacizumab therapy. She was admitted to the hospital to control the CHF. The CHF alleviated after 4 weeks’ treatment. She was then discharged from the hospital with oral cardiotonics and diuretics. Three months later, the LVEF recovered to 69%. The LVEF was 56% and 60% at six months and one year after her hospital discharge, respectively. This outcome implied that the cardiac toxicity in patients who were treated with bevacizumab might be reversible. This was also reported in another paper [26].

## Conclusion

In this small population, the addition of the bevacizumab to the standard R-CHOP regimen caused bevacizumab-specific toxicities. Although the gum bleeding, epistaxis and proteinuria were mild, the congestive heart failure was a more severe. Early detection and personalized management may improve clinical outcome tolerance. This implies that the cardiac toxicity of the A-R-CHOP regimen should be monitored closely.

## Competing interests

The authors declare that they have no competing interests.

## Authors’ contributions

Enrolled the patients: YS, JZ, WZ, ZF, YX, XW, NL, MT, LP, ZY,WL, LD, CZ. Collected data: ZF. Analyzed data: YS, JZ, WZ, ZF. Drafted the manuscript: ZF. Revised the manuscript: YS, JZ, WL, ND. All authors read and approved the final manuscript.
